# Supplementary figures and images for: What can drawings tell us about children’s perceptions of nature?
Source: PLoS One. 2023 Jul 5;18(7):e0287370. doi: 10.1371/journal.pone.0287370 (PMC10321616; doi:10.1371/journal.pone.0287370)

**S1 Fig**

**
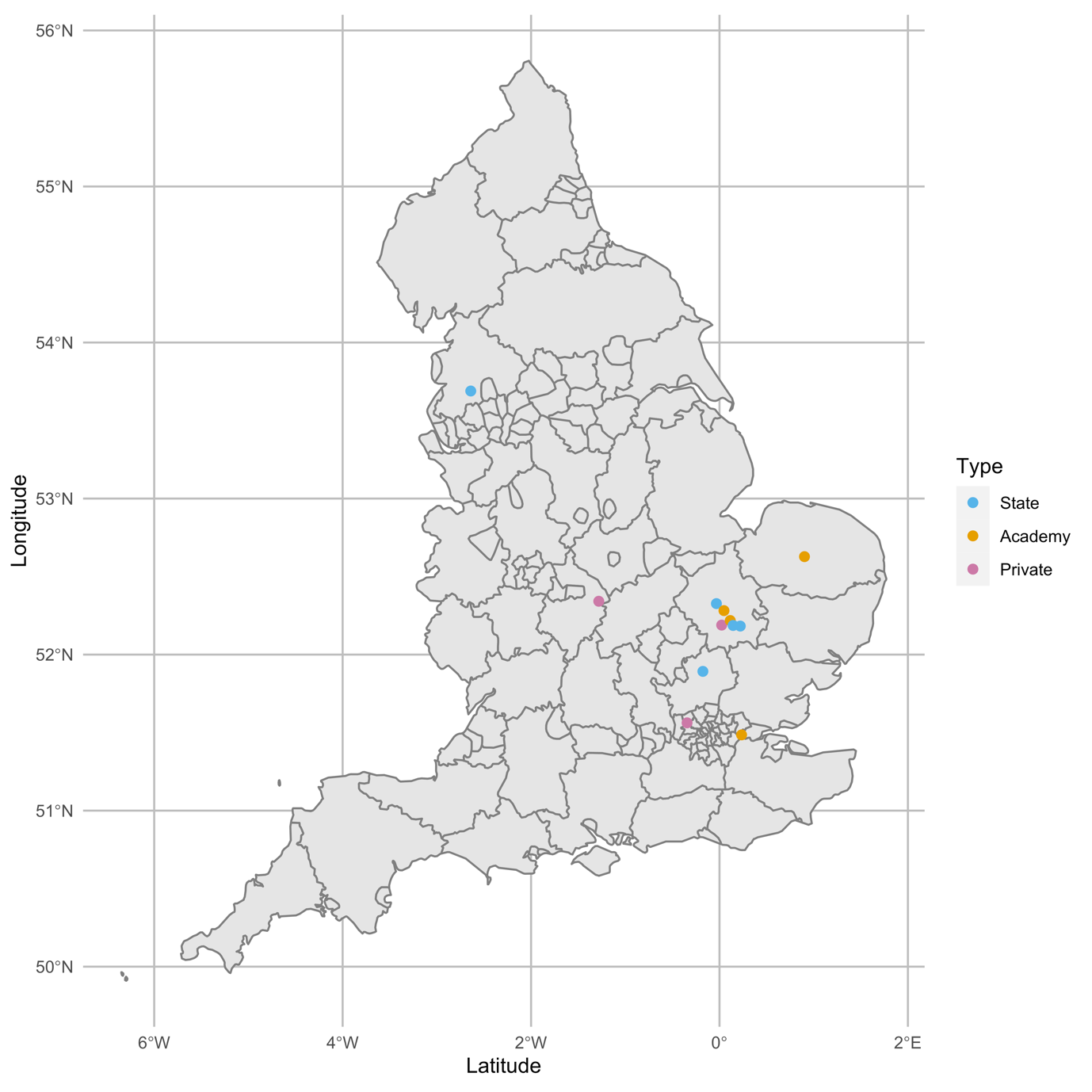
**

Supplement: S1 Fig — Locations of primary schools across England from which drawings were collected, coloured by school type. Each dot represents one school (n = 12). The private school in the main cluster of points has been manually moved 0.1 degrees west, so that all points are visible. (DOCX) [file pone.0287370.s001.docx]
