# Supplementary material for: What can drawings tell us about children’s perceptions of nature?
Source: PLoS One. 2023 Jul 5;18(7):e0287370. doi: 10.1371/journal.pone.0287370 (PMC10321616; doi:10.1371/journal.pone.0287370)
Supplement: S1 Table — Table showing the distribution of children’s drawings (n = 401) across ages and schools (n = 12), including school location and type. (DOCX) [file pone.0287370.s002.docx]

**S2 Table**

| **Type** | **Local authority** | **Children’s age** | **No. of drawings** |
| --- | --- | --- | --- |
| State | Cambridge | 7-8 years | 29 |
| State | Chorley | 10-11 years | 34 |
| State | Huntingdonshire | 8-9 years | 27 |
| State | South Cambridgeshire | 9-11 years | 80 |
| State | Stevenage | 9-10 years | 72 |
| Academy | Breckland | 7-8 years | 30 |
| Academy | Cambridge | 9-10 years | 24 |
| Academy | South Cambridgeshire | 7-10 years | 12 |
| Academy | Thurrock | 7-8 years | 29 |
| Private | Cambridge | 9-10 years | 17 |
| Private | Harrow | 10-11 years | 9 |
| Private | Warwick | 8-11 years | 38 |
|  |  |  |  |
