# Supplementary material for: What can drawings tell us about children’s perceptions of nature?
Source: PLoS One. 2023 Jul 5;18(7):e0287370. doi: 10.1371/journal.pone.0287370 (PMC10321616; doi:10.1371/journal.pone.0287370)
Supplement: S2 Table — Table showing all terms used in the labels and captions of children’s drawings, detailing assigned categories, whether or not a term’s presence was counted towards the species richness of a drawing, and the count and ID level for each term. Despite technically being the same species, ‘Pony’ and ‘Horse’ are both counted towards species richness because, to a child, these are different animals. ‘Mushroom’ is included in the category ‘Other Plants’ since it is the only fungal representative, and it appeared in just three drawings. (DOCX) [file pone.0287370.s003.docx]

## **S5 Table**

| **Category**  ***(% drawings containing at least one member)*** | **Term used as label or in caption** | **Included towards species richness count?** | **Taxonomic ID level** | **No. drawings containing term**  **(*n* = 401)** | **% drawings containing term** |
| --- | --- | --- | --- | --- | --- |
| ***Animals*** *(Total: 123 terms)* | | | | | |
| General *(57.6%)* | 1. Birds | n | Class | 195 | 48.6 |
| General | 1. Bugs | n | Order | 27 | 6.7 |
| General | 1. Creepy crawlies | n | Kingdom | 1 | 0.2 |
| General | 1. Fish | n | Phylum | 20 | 5.0 |
| General | 1. Insects | n | Class | 24 | 6.0 |
| General | 1. Mammals | n | Class | 10 | 2.5 |
| General | 1. Minibeasts | n | Kingdom | 3 | 0.7 |
| Domestic Mammal *(49.6%)* | 1. Cat | y | Species | 106 | 26.4 |
| Domestic Mammal | 1. Dog | y | Species | 68 | 17.0 |
| Domestic Mammal | 1. Gerbil | y | Species | 1 | 0.2 |
| Domestic Mammal | 1. Guinea pig | y | Species | 9 | 2.2 |
| Domestic Mammal | 1. Hamster | y | Family | 2 | 0.5 |
| Domestic Mammal | 1. Horse | y | Species | 2 | 0.5 |
| Domestic Mammal | 1. Pig | y | Species | 2 | 0.5 |
| Domestic Mammal | 1. Pony | y | Species | 2 | 0.5 |
| Domestic Mammal | 1. Rabbit | y | Species | 56 | 14.0 |
| Domestic Mammal | 1. Sheep | y | Species | 1 | 0.2 |
| Wild Mammal *(57.1%)* | 1. Badger | y | Species | 3 | 0.7 |
| Wild Mammal | 1. Bat | y | Order | 8 | 2.0 |
| Wild Mammal | 1. Deer | y | Family | 23 | 5.7 |
| Wild Mammal | 1. Flying squirrel | y | Family | 1 | 0.2 |
| Wild Mammal | 1. Fox | y | Species | 56 | 14.0 |
| Wild Mammal | 1. Grey squirrel | y | Species | 2 | 0.5 |
| Wild Mammal | 1. Hare | y | Species | 1 | 0.2 |
| Wild Mammal | 1. Hedgehog | y | Species | 79 | 19.7 |
| Wild Mammal | 1. Hippo | y | Species | 1 | 0.2 |
| Wild Mammal | 1. Lion | y | Species | 1 | 0.2 |
| Wild Mammal | 1. Mole | y | Species | 20 | 5.0 |
| Wild Mammal | 1. Mouse | y | Genus | 25 | 6.2 |
| Wild Mammal | 1. Muntjac deer | y | Genus | 6 | 1.5 |
| Wild Mammal | 1. Rat | y | Genus | 16 | 4.0 |
| Wild Mammal | 1. Red squirrel | y | Species | 1 | 0.2 |
| Wild Mammal | 1. Shrew | y | Order | 1 | 0.2 |
| Wild Mammal | 1. Squirrel | y | Genus | 120 | 29.9 |
| Wild Mammal | 1. Tiger | y | Species | 1 | 0.2 |
| Wild Mammal | 1. Wildcat | y | Species | 3 | 0.7 |
| Garden Bird *(24.9%)* | 1. Blackbird | y | Species | 24 | 6.0 |
| Garden Bird | 1. Blue tit | y | Species | 27 | 6.7 |
| Garden Bird | 1. Chaffinch | y | Species | 1 | 0.2 |
| Garden Bird | 1. Collared dove | y | Species | 1 | 0.2 |
| Garden Bird | 1. Crow | y | Species | 15 | 3.7 |
| Garden Bird | 1. Dove | y | Family | 2 | 0.5 |
| Garden Bird | 1. Dunnock | y | Species | 1 | 0.2 |
| Garden Bird | 1. Goldfinch | y | Species | 4 | 1.0 |
| Garden Bird | 1. Great tit | y | Species | 4 | 1.0 |
| Garden Bird | 1. Greenfinch | y | Species | 2 | 0.5 |
| Garden Bird | 1. Green woodpecker | y | Genus | 1 | 0.2 |
| Garden Bird | 1. Jackdaw | y | Species | 1 | 0.2 |
| Garden Bird | 1. Jay | y | Species | 2 | 0.5 |
| Garden Bird | 1. Long-tailed tit | y | Species | 2 | 0.5 |
| Garden Bird | 1. Magpie | y | Species | 9 | 2.2 |
| Garden Bird | 1. Pigeon | y | Family | 53 | 13.2 |
| Garden Bird | 1. Robin | y | Species | 39 | 9.7 |
| Garden Bird | 1. Sparrow | y | Genus | 6 | 1.5 |
| Garden Bird | 1. Starling | y | Species | 1 | 0.2 |
| Garden Bird | 1. Thrush | y | Genus | 1 | 0.2 |
| Garden Bird | 1. Wagtail | y | Genus | 1 | 0.2 |
| Garden Bird | 1. Woodpecker | y | Family | 4 | 1.0 |
| Garden Bird | 1. Wren | y | Species | 2 | 0.5 |
| Other Bird *(13.7%)* | 1. Barn owl | y | Species | 1 | 0.2 |
| Other Bird | 1. Bird of prey | y | Class | 4 | 1.0 |
| Other Bird | 1. Chicken | y | Species | 9 | 2.2 |
| Other Bird | 1. Duck | y | Family | 15 | 3.7 |
| Other Bird | 1. Eagle | y | Family | 1 | 0.2 |
| Other Bird | 1. Goose | y | Family | 1 | 0.2 |
| Other Bird | 1. Gull | y | Family | 1 | 0.2 |
| Other Bird | 1. Heron | y | Family | 1 | 0.2 |
| Other Bird | 1. House martin | y | Species | 1 | 0.2 |
| Other Bird | 1. Kestrel | y | Species | 1 | 0.2 |
| Other Bird | 1. Moorhen | y | Species | 2 | 0.5 |
| Other Bird | 1. Owl | y | Order | 11 | 2.7 |
| Other Bird | 1. Parrot | y | Order | 1 | 0.2 |
| Other Bird | 1. Partridge | y | Family | 6 | 1.5 |
| Other Bird | 1. Peacock | y | Species | 2 | 0.5 |
| Other Bird | 1. Peregrine falcon | y | Species | 1 | 0.2 |
| Other Bird | 1. Pheasant | y | Species | 10 | 2.5 |
| Other Bird | 1. Red kite | y | Species | 2 | 0.5 |
| Other Bird | 1. Skylark | y | Species | 1 | 0.2 |
| Other Bird | 1. Swallow | y | Species | 1 | 0.2 |
| Other Bird | 1. Swan | y | Genus | 2 | 0.5 |
| Other Bird | 1. Swift | y | Species | 3 | 0.7 |
| Herpetofauna *(15.7%)* | 1. Frog | y | Order | 51 | 12.7 |
| Herpetofauna | 1. Grass snake | y | Species | 9 | 2.2 |
| Herpetofauna | 1. Newt | y | Family | 1 | 0.2 |
| Herpetofauna | 1. Snake | y | Order | 3 | 0.7 |
| Herpetofauna | 1. Tadpole | y | Class | 4 | 1.0 |
| Herpetofauna | 1. Toad | y | Order | 3 | 0.7 |
| Herpetofauna | 1. Tortoise | y | Order | 3 | 0.7 |
| Insect *(52.9%)* | 1. Ant | y | Family | 116 | 28.9 |
| Insect | 1. Aphid | y | Order | 1 | 0.2 |
| Insect | 1. Bee | y | Family | 102 | 25.4 |
| Insect | 1. Beetle | y | Order | 11 | 2.7 |
| Insect | 1. Blackfly | y | Family | 1 | 0.2 |
| Insect | 1. Black garden ant | y | Species | 1 | 0.2 |
| Insect | 1. Bumblebee | y | Genus | 5 | 1.2 |
| Insect | 1. Butterfly | y | Order | 64 | 16.0 |
| Insect | 1. Caterpillar | y | Order | 23 | 5.7 |
| Insect | 1. Cricket | y | Order | 1 | 0.2 |
| Insect | 1. Dragonfly | y | Order | 10 | 2.5 |
| Insect | 1. Fly | y | Order | 23 | 5.7 |
| Insect | 1. Grasshopper | y | Order | 7 | 1.7 |
| Insect | 1. Greenfly | y | Species | 3 | 0.7 |
| Insect | 1. Hornet | y | Genus | 1 | 0.2 |
| Insect | 1. Ladybird | y | Family | 35 | 8.7 |
| Insect | 1. Larvae | y | Class | 1 | 0.2 |
| Insect | 1. Mayfly | y | Order | 2 | 0.5 |
| Insect | 1. Midge | y | Order | 1 | 0.2 |
| Insect | 1. Mosquito | y | Family | 1 | 0.2 |
| Insect | 1. Moth | y | Order | 5 | 1.2 |
| Insect | 1. Red ant | y | Species | 1 | 0.2 |
| Insect | 1. Shield bug | y | Order | 1 | 0.2 |
| Insect | 1. Stag beetle | y | Family | 3 | 0.7 |
| Insect | 1. Violet ground beetle | y | Species | 1 | 0.2 |
| Insect | 1. Wasp | y | Order | 30 | 7.5 |
| Insect | 1. Yellow meadow ant | y | Species | 1 | 0.2 |
| Other Invertebrate *(50.9%)* | 1. Arachnids | y | Class | 2 | 0.5 |
| Other Invertebrate | 1. Centipede | y | Class | 9 | 2.2 |
| Other Invertebrate | 1. Millipede | y | Class | 6 | 1.5 |
| Other Invertebrate | 1. Slug | y | Class | 51 | 12.7 |
| Other Invertebrate | 1. Snail | y | Class | 94 | 23.4 |
| Other Invertebrate | 1. Spider | y | Order | 97 | 24.2 |
| Other Invertebrate | 1. Woodlouse | y | Order | 37 | 9.2 |
| Other Invertebrate | 1. Worm | y | Phylum | 103 | 25.7 |
| ***Plants*** *(Total: 56 terms)* | | | | | |
| General *(79.6%)* | 1. Berries | n | None | 1 | 0.2 |
| General | 1. Blossom | n | None | 1 | 0.2 |
| General | 1. Bush/Shrub/Hedge | n | None | 93 | 23.2 |
| General | 1. Evergreen tree | n | None | 1 | 0.2 |
| General | 1. Flower | n | None | 112 | 27.9 |
| General | 1. Fruit tree | n | None | 12 | 3.0 |
| General | 1. Plant | n | None | 49 | 12.2 |
| General | 1. Tree | n | None | 247 | 61.6 |
| General | 1. Weed | n | None | 2 | 0.5 |
| Tree *(6.5%)* | 1. Apple tree | y | Species | 12 | 3.0 |
| Tree | 1. Cherry tree | y | Genus | 6 | 1.5 |
| Tree | 1. Crab apple tree | y | Genus | 2 | 0.5 |
| Tree | 1. Cypress | y | Family | 1 | 0.2 |
| Tree | 1. Olive tree | y | Species | 3 | 0.7 |
| Tree | 1. Palm tree | y | Family | 1 | 0.2 |
| Tree | 1. Pear tree | y | Genus | 7 | 1.7 |
| Tree | 1. Plum tree | y | Genus | 2 | 0.5 |
| Flower *(2.7%)* | 1. Daffodil | y | Genus | 1 | 0.2 |
| Flower | 1. Dandelion | y | Genus | 1 | 0.2 |
| Flower | 1. Lavender | y | Genus | 2 | 0.5 |
| Flower | 1. Lily | y | Genus | 1 | 0.2 |
| Flower | 1. Poppy | y | Family | 1 | 0.2 |
| Flower | 1. Rose | y | Genus | 5 | 1.2 |
| Flower | 1. Snowdrop | y | Genus | 1 | 0.2 |
| Flower | 1. Sunflower | y | Genus | 1 | 0.2 |
| Flower | 1. Tulip | y | Genus | 1 | 0.2 |
| Crop *(5.2%)* | 1. Blackberries | y | Genus | 2 | 0.5 |
| Crop | 1. Blueberries | y | Genus | 1 | 0.2 |
| Crop | 1. Broccoli | y | Species | 1 | 0.2 |
| Crop | 1. Carrots | y | Species | 4 | 1.0 |
| Crop | 1. Chilli | y | Genus | 1 | 0.2 |
| Crop | 1. Grapes | y | Genus | 1 | 0.2 |
| Crop | 1. Green beans | y | Species | 1 | 0.2 |
| Crop | 1. Herbs | n | None | 3 | 0.7 |
| Crop | 1. Lettuce | y | Species | 1 | 0.2 |
| Crop | 1. Mint | y | Genus | 1 | 0.2 |
| Crop | 1. Peas | y | Species | 1 | 0.2 |
| Crop | 1. Potatoes | y | Species | 1 | 0.2 |
| Crop | 1. Raspberries | y | Genus | 2 | 0.5 |
| Crop | 1. Rhubarb | y | Species | 1 | 0.2 |
| Crop | 1. Rosemary | y | Species | 1 | 0.2 |
| Crop | 1. Strawberries | y | Species | 7 | 1.7 |
| Crop | 1. Thyme | y | Genus | 1 | 0.2 |
| Crop | 1. Tomatoes | y | Species | 1 | 0.2 |
| Crop | 1. Vegetables | n | None | 5 | 1.2 |
| Other Plants *(60.6%)* | 1. Bamboo | y | Family | 1 | 0.2 |
| Other Plants | 1. Bramble | y | Genus | 1 | 0.2 |
| Other Plants | 1. Fern | y | Class | 1 | 0.2 |
| Other Plants | 1. Grass | y | Family | 237 | 59.1 |
| Other Plants | 1. Heather | y | Family | 1 | 0.2 |
| Other Plants | 1. Holly | y | Genus | 1 | 0.2 |
| Other Plants | 1. Ivy | y | Genus | 2 | 0.5 |
| Other Plants | 1. Mushroom | y | Kingdom | 3 | 0.7 |
| Other Plants | 1. Pampas grass | y | Family | 1 | 0.2 |
| Other Plants | 1. Reeds | y | None | 1 | 0.2 |
| Other Plants | 1. Rhododendron | y | Genus | 1 | 0.2 |
