# Supplementary material for: What can drawings tell us about children’s perceptions of nature?
Source: PLoS One. 2023 Jul 5;18(7):e0287370. doi: 10.1371/journal.pone.0287370 (PMC10321616; doi:10.1371/journal.pone.0287370)
Supplement: S3 Table — Table showing the numbers and proportions of children’s drawings (n = 401) that contained at least one representative of each group (see S2 Table for a full list of terms and categories). (DOCX) [file pone.0287370.s004.docx]

**S6 Table**

| **Group**  ***(List of categories and extra terms included)*** | **No. drawings (*n* = 401) containing at least one mention** | **% drawings containing at least one mention** |
| --- | --- | --- |
| Mammal  *(incl. all terms in the categories ‘Domestic Mammals’ and ‘Wild Mammals’, and the ‘General’ term ‘Mammal’)* | 323 | 80.5 |
| Bird  *(incl. all terms in the categories ‘Garden Birds’ and ‘Other Birds’, and the ‘General’ term ‘Bird’)* | 275 | 68.6 |
| Herpetofauna  *(incl. all terms in the category ‘Herpetofauna’)* | 63 | 15.7 |
| Insect  *(incl. all terms in the category ‘Insects’ and the ‘General’ terms ‘Bug’ and ‘Insect’)* | 219 | 54.6 |
| Other invertebrate  *(incl. all terms in the category ‘Other Invertebrates’ and the ‘General’ terms ‘Creepy crawlies’ and ‘Minibeasts’)* | 206 | 51.4 |
| ‘General’ animal term(s) only | 11 | 2.7 |
| Tree  *(incl. all terms in the category ‘Trees’ and the ‘General’ terms ‘Evergreen tree’, ‘Fruit tree’ and ‘Tree’)* | 269 | 67.1 |
| Flower  *(incl. all terms in the category ‘Flowers’ and the ‘General’ terms ‘Blossom’ and ‘Flower’)* | 121 | 30.2 |
| Crop  *(incl. all terms in the category ‘Crops’ and the ‘General’ term ‘Berries’)* | 21 | 5.2 |
| Other plant  *(incl. all terms in the category ‘Other Plants’ and the ‘General’ terms ‘Bush’, ‘Plant’ and ‘Weed’)* | 290 | 72.3 |
| ‘General’ plant term(s) only | 106 | 26.4 |
|  |  |  |
