# Supplementary material for: What can drawings tell us about children’s perceptions of nature?
Source: PLoS One. 2023 Jul 5;18(7):e0287370. doi: 10.1371/journal.pone.0287370 (PMC10321616; doi:10.1371/journal.pone.0287370)
Supplement: S4 Table — Table showing results of post-hoc analyses following a significant effect of school type in the parent model. For plant species richness, p values are the results of Tukey all-pair comparisons, adjusted via the Bonferroni correction, following a significant effect of school type in the GLMM. State school drawings had higher species richness than private school drawings. For animal community composition, p values are the results of pairwise comparisons via an analysis of deviance for the mGLM, adjusted for multiple comparisons via a free step-down resampling procedure. State school drawings contained more terms for invertebrates other than insects, and private school drawings contained more terms for garden birds. Significant p values are shown in bold. (DOCX) [file pone.0287370.s005.docx]

## **S7 Table**

| **Pairwise comparison** | **Adjusted *p* value** |
| --- | --- |
| ***Plant species richness*** | |
| State—Academy | 1 |
| State­—Private | **0.00611** |
| Private—Academy | 0.06489 |
| ***Animal community composition*** | |
| State—Academy | **0.005** |
| State­—Private | **0.006** |
| Private—Academy | **0.006** |
