# Supplementary material for: What can drawings tell us about children’s perceptions of nature?
Source: PLoS One. 2023 Jul 5;18(7):e0287370. doi: 10.1371/journal.pone.0287370 (PMC10321616; doi:10.1371/journal.pone.0287370)
Supplement: S5 Table — Results of chi-square tests of independence on differences in taxonomic resolution of animal terms in each category by school type. (DOCX) [file pone.0287370.s006.docx]

## **S8 Table**

| **Category** | *χ*² | **df** | ***p* value** |
| --- | --- | --- | --- |
| Domestic mammal | 0.052458 | 2 | 0.9741 |
| Wild mammal | 0.54349 | 2 | 0.7621 |
| Garden bird | 0.054229 | 2 | 0.9732 |
| Other bird | 0.25421 | 2 | 0.8806 |
| Herpetofauna | 0.092517 | 2 | 0.9548 |
| Insect | 2.8187 | 2 | 0.2443 |
| Other invertebrate | NaN | 2 | NA |
