# Supplementary material for: What can drawings tell us about children’s perceptions of nature?
Source: PLoS One. 2023 Jul 5;18(7):e0287370. doi: 10.1371/journal.pone.0287370 (PMC10321616; doi:10.1371/journal.pone.0287370)
Supplement: S6 Table — Results of chi-square tests of independence on differences in taxonomic resolution of plant terms in each category by school type. (DOCX) [file pone.0287370.s007.docx]

## **S9 Table**

| **Category** | *χ*² | **df** | ***p* value** |
| --- | --- | --- | --- |
| Tree | 0.66349 | 2 | 0.7177 |
| Flower | NaN | 2 | NA |
| Crop | 0.91926 | 2 | 0.6315 |
| Other | NaN | 2 | NA |
