# Supplementary material for: What can drawings tell us about children’s perceptions of nature?
Source: PLoS One. 2023 Jul 5;18(7):e0287370. doi: 10.1371/journal.pone.0287370 (PMC10321616; doi:10.1371/journal.pone.0287370)
Supplement: S1 Appendix — Worksheet with instructions given to primary-school children. Font size and box dimensions have been reduced in order to fit within document margins. (DOCX) [file pone.0287370.s008.docx]

## **S3 Appendix**

Please draw and label a picture of your garden or local park showing the animals you think live there.

Tell us a little bit about what you have drawn below.

I have drawn _______________________________________________ __________________________________________________________ __________________________________________________________
