# Supplementary material for: What can drawings tell us about children’s perceptions of nature?
Source: PLoS One. 2023 Jul 5;18(7):e0287370. doi: 10.1371/journal.pone.0287370 (PMC10321616; doi:10.1371/journal.pone.0287370)
Supplement: S2 Appendix — Information provided to parents or guardians of children before the study. (DOCX) [file pone.0287370.s009.docx]

## **S4 Appendix**

**Information for Potential Child Participants and their Families**

Before you decide to give consent for your child to take part in this study, it is important for you to understand why the research is being done and what it will involve. Please take time to read the following information carefully and discuss it with others if you wish. Please contact Kate Howlett (kh557@cam.ac.uk) at the University of Cambridge Museum of Zoology if there is anything that is not clear or if you would like more information. Please take time to decide whether or not you wish your child to take part.

**What is the research about?**

We are investigating the green space that UK primary school children have access to at school. We are interested in the importance of these spaces for children's learning and wellbeing, and in how they affect children’s relationship with and awareness of nature. These drawings will help us answer these questions.

**Why has my child been asked to participate?**

We hope to collect responses from as a wide a range of children as possible, so individuals have not been selected on any particular basis.

**What does the study entail?**

After giving your consent for your child to take part, your child will be given a worksheet to complete in class, under supervision by their teacher, along with their classmates. This worksheet will ask them to draw a picture of their garden or local park, labelling all the animals they think live there. At the bottom of the sheet, there will be a couple of lines in which they can tell us a bit more about what they have drawn. The drawings will then be collected in by their teacher and passed onto the researchers for analysis.

**Does my child have to take part?**

No. You do not have to give consent to your child taking part in this research. But, if you did consent, researchers know from past experience that most children have enjoyed the task.

**Will participation be confidential?**

Yes. No personal data will be collected at any point, and your child will not be identifiable from their drawings. All information collected will remain strictly confidential and will not be shared beyond the small research team of two. Drawings will be linked to each child’s school by a numerical code, the details of which will be stored in a password-protected file, accessible only by the immediate research team.

General guidance on how the University uses personal data can be found at https://www.information-compliance.admin.cam.ac.uk/data-protection/research-participant-data.

Your child will not be identified in any report or publication. Results will be presented at conferences and written up in peer-reviewed journal papers. Results will usually be presented in terms of groups of individuals. If data from any individual are presented, the data will be totally anonymous, without any means of identifying the individual(s) involved.

**What happens if I change my mind?**

Taking part is entirely voluntary, and refusal or withdrawal will involve no penalty or loss, either now or at any point in the future. You are free to contact the research team at kh557@cam.ac.uk to withdraw your consent at any point in the future, up until three months after taking part.

This research is funded by the Natural Environment Research Council, and this project has been reviewed by the Psychology Research Ethics Committee of the University of Cambridge.

**Consent Form**

Please tick each box below to acknowledge that you have read, understood and agreed to the following statements, then sign and date below.

☐ I confirm that I have read and understood the Information for Potential Child Participants and their Families.

☐ I understand that I can contact the research team via kh557@cam.ac.uk at any point to ask for more information.

☐ I understand that all information collected will remain confidential and that all efforts will be made to ensure my child cannot be identified.

☐ I agree that data gathered in this study may be stored anonymously and securely and may be used for future research.

☐ I understand that my consent is voluntary and that I am free to withdraw my consent at any time without giving a reason, up until three months after taking part, by contacting kh557@cam.ac.uk.

☐ I give consent for my child to take part.

Name: ________________________________

Signed: ________________________________

Child’s name: ________________________________

Date:_____________
