# Supplementary material for: What can drawings tell us about children’s perceptions of nature?
Source: PLoS One. 2023 Jul 5;18(7):e0287370. doi: 10.1371/journal.pone.0287370 (PMC10321616; doi:10.1371/journal.pone.0287370)
Supplement: Attachment — Submitted filename: Response to Reviewers.docx [file pone.0287370.s011.docx]

PONE-D-22-22596R1

What can drawings tell us about children’s perceptions of nature?

PLOS ONE

Dear Dr Silva,

Thank you for coordinating the review process for our manuscript and to all reviewers for providing such helpful comments and suggestions, which have greatly improved the article.

We have responded to the reviewers’ comments point-by-point below.

We have also addressed the Journal Requirements as follows:

Journal Requirements: Please review your reference list to ensure that it is complete and correct. If you have cited papers that have been retracted, please include the rationale for doing so in the manuscript text, or remove these references and replace them with relevant current references. Any changes to the reference list should be mentioned in the rebuttal letter that accompanies your revised manuscript. If you need to cite a retracted article, indicate the article’s retracted status in the References list and also include a citation and full reference for the retraction notice.

Response: I have checked all references and the reference list, and I am happy that this is correct.

In addition to this rebuttal letter, we have provided a marked-up copy of the manuscript highlighting changes made and an unmarked version without tracked changes, as requested.

We look forward to hearing from you.

Kind regards,

Kate Howlett

Response to Reviewers' Comments to the Author:

Reviewer #5: Dear authors, I congratulate you on this beautiful study. The topic is extremely relevant and important given the threats we face as a society for the conservation of biodiversity. In general, the text is very coherent with all the necessary elements to report the study and provide the necessary information. The analyses are well done and the results are organised and presented in a coherent manner. A few adjustments are relevant, both to conform to the journal's standards and to help the reader understand the text.

- Thank you for your positive comments on our paper and support of our work. Thank you also for your below suggestions, which have definitely improved it.

Abstract: The journal standard requires an abstract of up to 300 words. In the manuscript the abstract is 502. I suggest you look at <https://chemistrycommunity.nature.com/posts/43071-how-to-write-an-abstract>.

- Thank you for highlighting this. I have now cut the abstract (see tracked changes for lines 21-263) to abide by the journal’s word limit.

Line 293 has an incomplete sentence.

- Thank you for pointing this out. This was a result of changing the referencing style. I have now corrected this (see tracked changes in line 505).

Line 453 gives a value of "0%". This definition looks strange in the text and it is better to say that there was no identification until species for invertebrates other than insects.

- Thank you for suggesting this. I have incorporated this (see tracked changes in lines 665-666), and the sentence now reads much better.
